# Laparoscopic versus ultrasound-guided transversus abdominis plane block for postoperative pain management in minimally invasive colorectal surgery: a meta-analysis protocol

**DOI:** 10.3389/fonc.2023.1080327

**Published:** 2023-05-22

**Authors:** Wenming Yang, Tao Yuan, Zhaolun Cai, Qin Ma, Xueting Liu, Hang Zhou, Siyuan Qiu, Lie Yang

**Affiliations:** ^1^ Division of Gastrointestinal Surgery, Department of General Surgery, West China Hospital, Sichuan University, Chengdu, China; ^2^ Department of Anesthesiology, West China Hospital, Sichuan University, Chengdu, China; ^3^ Gastric Cancer Center, Department of General Surgery, West China Hospital, Sichuan University, Chengdu, China; ^4^ Department of Medical Discipline Construction, West China Hospital, Sichuan University, Chengdu, China

**Keywords:** transversus abdominis plane block, postoperative pain management, minimally invasive, colorectal surgery, data synthesis

## Abstract

**Introduction:**

Transversus abdominis plane block (TAPB) is now commonly administered for postoperative pain control and reduced opioid consumption in patients undergoing major colorectal surgeries, such as colorectal cancer, diverticular disease, and inflammatory bowel disease resection. However, there remain several controversies about the effectiveness and safety of laparoscopic TAPB compared to ultrasound-guided TAPB. Therefore, the aim of this study is to integrate both direct and indirect comparisons to identify a more effective and safer TAPB approach.

**Materials and methods:**

Systematic electronic literature surveillance will be performed in the PubMed, Embase, Cochrane Central Register of Controlled Trials (CENTRAL), and ClinicalTrials.gov databases for eligible studies through July 31, 2023. The Cochrane Risk of Bias version 2 (RoB 2) and Risk of Bias in Non-randomized Studies of Interventions (ROBINS-I) tools will be applied to scrutinize the methodological quality of the selected studies. The primary outcomes will include (1) opioid consumption at 24 hours postoperatively and (2) pain scores at 24 hours postoperatively both at rest and at coughing and movement according to the numerical rating scale (NRS). Additionally, the probability of TAPB-related adverse events, overall postoperative 30-day complications, postoperative 30-day ileus, postoperative 30-day surgical site infection, postoperative 7-day nausea and vomiting, and length of stay will be analyzed as secondary outcome measures. The findings will be assessed for robustness through subgroup analyses and sensitivity analyses. Data analyses will be performed using RevMan 5.4.1 and Stata 17.0. P value of less than 0.05 will be defined as statistically significant. The certainty of evidence will be examined *via* the Grading of Recommendations, Assessment, Development, and Evaluation (GRADE) working group approach.

**Ethics and dissemination:**

Owing to the nature of the secondary analysis of existing data, no ethical approval will be required. Our meta-analysis will summarize all the available evidence for the effectiveness and safety of TAPB approaches for minimally invasive colorectal surgery. High-quality peer-reviewed publications and presentations at international conferences will facilitate disseminating the results of this study, which are expected to inform future clinical trials and help anesthesiologists and surgeons determine the optimal tailored clinical practice for perioperative pain management.

**Systematic review registration:**

https://www.crd.york.ac.uk/PROSPERO/display_record.php?RecordID=281720, identifier (CRD42021281720).

## 1 Introduction

Recently, minimally invasive surgery (MIS) has been recommended to treat colorectal diseases, such as colorectal cancer, inflammatory bowel disease, and diverticular disease, because of its equivalent efficacy and improved functional recovery (1–3). Compared to traditional protocols, enhanced recovery after surgery (ERAS) pathways could significantly shorten the length of hospital stay (LOS) and reduce the healthcare costs without compromising surgical outcomes (4–6). Acute postoperative pain, however, remains the most common concern of ERAS. Meanwhile, regular administration of opioids is associated with postoperative ileus (POI), postoperative nausea and vomiting (PONV), delayed mobilization, acute urinary retention, and early-term somnolence and delirium (7). Despite the increasing popularity of the conception of opioid-sparing multimodal analgesia, consensus on optimal pain management after MIS is lacking.

Transversus abdominis plane block (TAPB) as a type of local anesthesia involves the injection of a local anesthetic between the transversus abdominis and internal oblique muscles to infiltrate the segmental nerves at the level of T8-L1 (8, 9). TAPB is now commonly employed during laparoscopic colorectal surgery and has proven to be effective in reducing postoperative opioid consumption (10). In the ERAS Society Guidelines for Perioperative Care in Elective Colorectal Surgery 2018, the use of TAPB is strongly recommended instead of epidural analgesia in colorectal MIS (11). Both laparoscopic (Lap-) and ultrasound-guided (US-) TAPB have allowed to reduce the risk of peritoneal penetration and facilitate the accurate identification of the tissue plane (12).

Currently, training in the use of ultrasonography among anesthesiologists is commonplace in tertiary referral centers (13). Characterized by ability to perform dynamic maneuvers and assess long segments of nerves, lack of radiation and contraindications, and portability, ultrasonography is recognized as one of the optimal imaging modalities for peripheral nerves (14), which contributes to its widespread application in perioperative nerve blocks. Conversely, due to additional human, time, and economic costs, techniques of ultrasound-guided nerve blocks might not be available in the primary hospitals. Furthermore, despite the guidance of ultrasound, procedure-related inadvertent visceral injury still should not be ignored (15, 16). Lap-TAPB can seemly be a potential alternative to reduce the waste of healthcare resource. Though visualization of laparoscopy minimizes intraperitoneal injection and visceral injury originated from peritoneal penetration, the precise positioning of the nerves and planes can be compromised by Lap-TAPB compared to US-TAPB (17).

The existing systematic reviews generally aimed to assess the differences between TAPB and no-TAPB locoregional analgesia or placebo control in colorectal surgery (18–21). Focusing on not all colorectal MIS but only laparoscopic colorectal surgery, a recently published meta-analysis cannot provide a convincing conclusion owing to the small sample size (3 studies, 219 patients) (22). Above all, there remain several controversies about the effectiveness and safety of Lap-TAPB compared with US-TAPB, and high-quality evidence is needed to guide individualized clinical practice (23–25). We hypothesize that surgeon-performed Lap-TAPB would be non-inferior to anesthesiologist-delivered US-TAPB. To verify this, we conduct the present meta-analysis to compare the effectiveness and safety of the two specific TAPB approaches for postoperative analgesia in colorectal MIS.

## 2 Materials and methods

On October 26, 2021, the present meta-analysis protocol was prospectively registered at the International Prospective Register of Systematic Reviews (PROSPERO) (registration ID: CRD42021281720). Besides, the protocol follows the Preferred Reporting Items for Systematic Reviews and Meta-Analysis Protocols (PRISMA-P) 2015 checklist (Supplementary material) (26). The main text of our future meta-analysis will adhere to the Preferred Reporting Items for Systematic Reviews and Meta-Analyses (PRISMA) guidelines and the Cochrane Collaboration’s standardized methodology (27, 28).

### 2.1 Eligibility criteria

#### 2.1.1 Inclusion criteria

Detailed inclusion criteria will be developed using the PICOS description model (participants, intervention, controls, outcome measures, and study design) (29).

##### 2.1.1.1 Type of participants (P)

Patients with cancer, inflammatory bowel disease, diverticular disease, or other diseases scheduled to undergo colorectal resection MIS (including laparoscopic, hand-assisted laparoscopic, robot-assisted, and trans-anal) will be included in this study. Other restrictions consist of age (≥ 18 years old) and American Society of Anesthesiologists physical status score (I - III).

##### 2.1.1.2 Type of interventions (I)

Lap-TAPB performed by surgeons at the beginning or end of surgeries is administered by a traditional transcutaneous or intraperitoneal approach as the intervention. Under laparoscopic guidance, a bilateral TAPB with the “double pops” technique is performed using a total of 40 mL of local anesthetic. Particularly, a needle of 18 gauge is inserted under direct vision at the midpoint of the midaxillary line between the iliac crest and the lower costal margin, and then 2 mL of normal saline is injected to identify its position. The preplanned amount of local anesthetic will be injected at the same point after a bulge formation as a result of the internally pushed transversus abdominis muscle and peritoneum. The contralateral abdominal wall is treated with the same technique.

##### 2.1.1.3 Type of controls (C)

US-TAPB delivered by anesthesiologists prior to surgery was set as the control.

##### 2.1.1.4 Type of outcomes (O)

###### 2.1.1.4.1 Primary outcomes

• opioid consumption at 24 hours postoperatively;
• pain scores at 24 hours postoperatively both at rest and at coughing and movement according to the numerical rating scale (NRS).

###### 2.1.1.4.2 Secondary outcomes

• TAPB-related adverse events;
• overall postoperative 30-day complications (Clavien-Dindo classification grade II or higher) (30);
• postoperative 30-day POI;
• postoperative 30-day surgical site infection (SSI);
• postoperative 7-day PONV;
• LOS.

##### 2.1.1.5 Type of study design (S)

Prospective randomized controlled trials (RCTs), quasi-RCTs (e.g., participants were assigned to groups according to alternate days of the week), case-control studies and cohort studies in which at least one outcome of interest was evaluated will be included.

#### 2.1.2 Exclusion criteria

• patients undergoing only laparoscopic exploration, bypass or diverting ostomy, conversion to laparotomy, or without assignment to Lap-TAPB and US-TAPB groups;
• animal subjects;
• conference abstracts, case reports, letters, editorials, reviews, or non-controlled trials without available data;
• previously published literature or with overlapping data of the same clinical trial;
• studies with missing or insufficient data after contacting corresponding authors;
• literature in non-English languages.

### 2.2 Sources of information and strategies for searching

Systematic electronic literature surveillance will be conducted in the PubMed, Embase (OVID interface), and Cochrane Central Register of Controlled Trials (CENTRAL) databases utilizing TAPB-related text words and medical subject headings (MeSH) to obtain relevant studies published through July 31, 2023. All references of the included literature will be further retrieved to identify potential eligibility. Identifying some relevant studies through hand searching is planned to supplement searching whenever necessary. To review trials in progress, the ClinicalTrials.gov and the International Clinical Trials Registry Platform (ICTRP) databases will also be searched. The search will be confined to human subjects and the English language. The detailed and specific search strategy and syntax for the PubMed database are formulated (**Table 1**).

**Table 1:** The search strategy for the PubMed database.

Search	Search terms
**#1**	(colon*) OR (colonic) OR (right-colon) OR (left-colon) OR (col*) OR (colorectal) OR (colectomy) OR (mesocol*) OR (mesocolonic) OR (hemicol*) OR (hemicolectomy) OR (CME)
**#2**	(sigmoid*) OR (sigmoid colon) OR (rectosigmoid) OR (proctosigmoid)
**#3**	(rect*) OR (rectum) OR (rectal) OR (proct*) OR (proctectomy) OR (mesorect*) OR (mesorectum) OR (TME)
**#4**	(anal) OR (anus) OR (transanal) OR (trans-anal)
**#5**	(diverticul*) OR (diverticular) OR (diverticulitis) OR (diverticulosis) OR (diverticulitides)
**#6**	(volvulus) OR (intestinal volvulus) OR (colonic volvulus) OR (torsion)
**#7**	#1 OR #2 OR #3 OR #4 OR #5 OR #6
**#8**	(transversus abdominis plane block) OR (transversus abdominis plane) OR (TAPB) OR (TAP) OR (tapb) OR (tap)
**#9**	(laparoscop*) OR (laparoscopic) OR (hand-assisted laparoscopy) OR (peritoneoscopy) OR (peritoneoscopic)
**#10**	(robot*) OR (robotic) OR (robot-assisted)
**#11**	(minimally invasive) OR (MIS) OR (TAMIS) OR (natural orifice specimen extraction) OR (NOSE) OR (NOSES)
**#12**	#9 OR #10 OR #11
**#13**	#7 AND #8 AND #12

This search strategy will be modified as required for other electronic databases.

### 2.3 Study identification and data management

**Figure 1:**
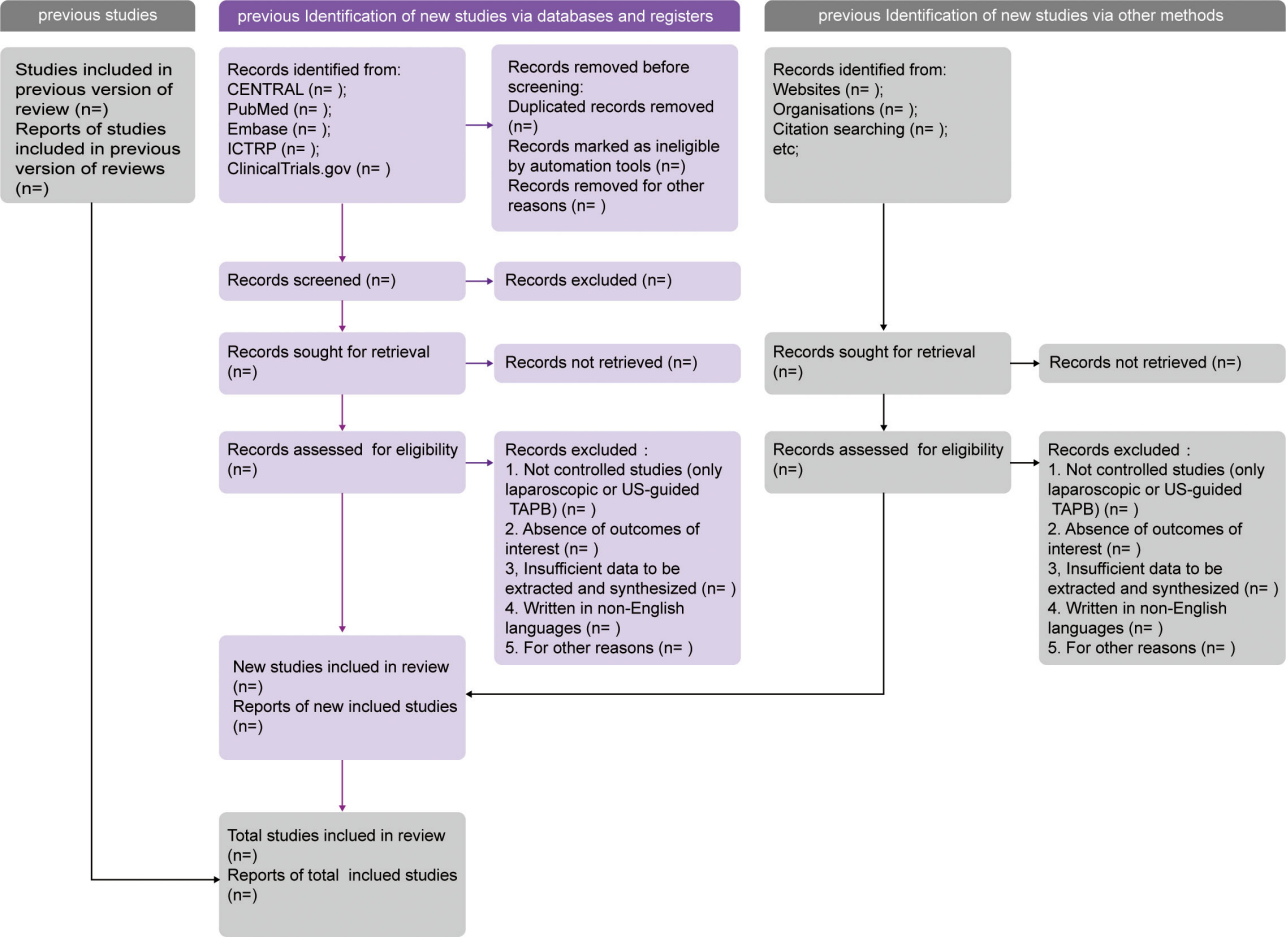
PRISMA 2020 flow diagram template of literature surveillance. PRISMA, Preferred Reporting Items for Systematic Reviews and Meta-Analyses. CENTRAL, the Cochrane Central Register of Controlled Trials. ICTRP, the International Clinical Trials Registry Platform.

Records obtained following the search strategy will be collected and imported in Mendeley software (RELX Group, Amsterdam, Netherlands). A team of three reviewers (WY, TY, and ZC) will independently screen the searched titles and abstracts against the eligibility criteria. For potentially eligible studies, full texts will be reviewed thoroughly. If there are a series of reports on one clinical trial, the latest publication containing the most sufficient data is suitable for inclusion. For further information on eligibility, corresponding authors of the studies will be contacted *via* e-mail whenever necessary. Thereafter, the above-mentioned three reviewers will reevaluate the entire texts post initial identification and document the reasons for some records to be excluded. All disputes among the three reviewers in this process will be settled *via* a consultation with a senior author (XL, LY). A structured PRISMA 2020 flowchart will be drawn to display the overview of the study identification procedure (**Figure 1**).

The review author pair (QM, HZ) will then extract general characteristic data of selected articles using a standardized electronic form designed by all authors (**Table 2**) for pooled analyses. A data set of first author’s name, publication year, country (or region), study design, study period, sample size, general characteristics of study population, TAPB technique and perioperative analgesia protocol, and all outcomes of interest will be collected from all eligible studies. Moreover, e-mails will be sent to the corresponding author to request adequate raw data in order to ensure the accuracy of the meta-analysis. If no effective response is received in 2 weeks, individual trials with missing data will be omitted from pooled analyses of the outcomes of interest. For high-quality management and synthesis, the cross-checked data will then be entered into Stata 17.0 software (StataCorp LLC, College Station, Texas, USA).

**Table 2:** Data extraction form.

Study details	
General information	
Trial registration number
First author
Year of publication
Country or region
Single centered or multicentered
Study time-frame/duration
Study eligibility	
Study design
Participants
Interventions
Controls
Outcome measures
Confounding variables
Include or exclude: Include □ Exclude □
Reason(s) for exclusion
Characteristics of included studies	
Sample size
Lap-TAPB group (dosage and regimen of local anesthetic)
US-guided TAPB group (dosage and regimen of local anesthetic)
Perioperative analgesia protocol (besides TAPB)
Data source
Age (mean or median) (years)
Gender distribution (male-female ratio)
Follow-up period (mean and range) (months)
Subgroups
Key conclusion(s)
Primary outcomes	
Opioid consumption at 24 hours postoperatively
Pain scores at 24 hours postoperatively
Secondary outcomes	
TAPB-related adverse event
Overall postoperative 30-day complication (≥ Clavien Dindo grade II)
Postoperative 30-day ileus (POI)
Postoperative 30-day surgical site infection (SSI)
Postoperative 7-day nausea and vomiting (PONV)
Length of postoperative hospital stay (LOS)

TAPB, transversus abdominis plane block; US, ultrasound.

### 2.4 Methodological quality assessment

The included studies will be meticulously evaluated by a team of three reviewers (WY, TY, and ZC) for methodological quality. The Cochrane Risk of Bias version 2 (RoB 2) tool is supposed to validate the risk of bias for included RCTs based on bias arising from the randomization process, bias due to deviations from intended interventions, bias due to missing outcome data, bias in measurement of the outcome, and bias in selection of the reported result (31). The RoB 2 Excel Tool (available at: https://www.riskofbias.info/welcome/rob-2-0-tool/current-version-of-rob-2 ) will be applied to implete RoB 2 for primary outcomes (31, 32). To quantify the risk of bias in each study, the Cochrane Handbook for Systematic Reviews of Interventions will be adopted (28). For non-randomized studies (NRSs), on the other hand, the Risk of Bias in Non-randomized Studies of Interventions (ROBINS-I) tool will be utilized (33). Due to potential risk of bias in the selected studies, the findings generated from this meta-analysis will be interpreted with caution.

### 2.5 Data synthesis and statistical analysis

Stata 17.0 and Review Manager 5.4.1 software (The Nordic Cochrane Centre, The Cochrane Collaboration, Copenhagen, Denmark) will be employed to conduct the present meta-analysis. For continuous outcomes (opioid consumption at 24 hours postoperatively, pain scores at 24 hours postoperatively, and LOS), pooled weighted (WMDs) or standardized mean differences (SMDs) with their respective 95% confidence intervals (CIs) will be calculated due to the uniformity of scales used in studies. In addition, pooled odds ratios (ORs) will be worked out with corresponding 95% CIs for dichotomous variables (TAPB-related adverse events, overall postoperative 30-day complications, POI, SSI, and PONV). As a way to summarize the findings across the studies, statistical significance level will be set at a p value of less than 0.05. Given that NRSs with large sample sizes could dominate and reverse the pooled effect estimates, data synthesis for the RCT group and NRS group will be performed separately. We will identify the statistical heterogeneity among studies using the χ² test and quantify it with Cochrane’s Inconsistency (*I²*)-statistic. We set 50% as a cutoff value, such that substantial heterogeneity is defined as *I²* exceeding 50% and/or p value less than 0.10. It is preferable to adopt a random-effect model (REM) if heterogeneity is considerable, or else a regular fixed-effect model (FEM) will be the alternative. Meanwhile, to investigate the potential sources of substantial heterogeneity, sensitivity analysis and significative subgroup analyses will be conducted. Whenever clinical heterogeneity is considerable, we will undertake a narrative review rather than a meta-analysis.

### 2.6 Sensitivity analysis and subgroup analyses

A leave-one-out sensitivity analysis will be carried out, which aims to verify the robustness of the primary outcomes’ measure effects regarding study design, sample size, heterogeneity qualities, and non-informative prior distribution for heterogeneity parameters.

The subgroup analyses listed below will be arranged if homogeneous outcomes are reported in multiple studies within the matched subgroups:

1) region/country: Asia versus other places;
2) type of MIS: laparoscopic versus robot-assisted;
3) natural orifice specimen extraction surgery: yes versus not;
4) neoadjuvant therapy: yes versus not;
5) NRSs with propensity-score matched analysis: yes versus not.

### 2.7 Publication bias

To ascertain the possibility of publication bias, we will firstly check whether the RCT protocol was published prior to the enrollment of patients for the study. Studies published after July 1, 2005 will be checked at the World Health Organization-affiliated ICTRP. The presence of outcome reporting bias (selective reporting of outcomes) will also be evaluated. The visual symmetry of a funnel plot will be considered as the primary predictor of publication bias when more than ten studies are included (34).

### 2.8 Confidence in cumulative evidence

In order to grade the certainty of evidence for each outcome, the Grading of Recommendations, Assessment, Development, and Evaluation (GRADE) working group approach will be chosen, which mainly contains the dimensions of study limitations, publication bias, imprecision, inconsistency, and indirectness (35). The evidence’s strength will be ranked as four levels: high (very confident that the effect estimate lies close to the true effect), moderate (moderately confident in the effect estimate), low (limitedly confident in the effect estimate), and very low (very little confident in the effect estimate) (36). In order to make the table and the process easier to be understood, all decisions to downgrade or upgrade the certainty of evidence will be accompanied by clear arguments in footnotes whenever necessary.

### 2.9 Ethical approval and dissemination

Owing to the nature of secondary analysis of existing data, there will be no patients involved in this study, and ethical approval will be not needed. High-quality peer-reviewed publications and presentations at international conferences will facilitate disseminating the results of this study.

## 3 Discussion

This protocol for a meta-analysis complies with the PRISMA-P guidelines. The subsequent meta-analysis will explore the effectiveness and safety of Lap-TAPB compared with US-TAPB on postoperative analgesia in colorectal MIS by summarizing the published studies. Furthermore, the meta-analysis is supposed to determine which subgroups benefit more from Lap-TAPB. The statistical analyses and other methodological processes will follow the PRISMA guidelines. The risk of bias will be examined *via* the Cochrane RoB 2 or ROBINS-I tool at the study level as well as the GRADE approach at the outcome level. Therefore, its findings are expected to build the foundation for future research and provide evidence-based tailored guidance on postoperative pain management for patients undergoing colorectal MIS.

## Data availability statement

The original contributions presented in the study are included in the article/**Supplementary Material**. Further inquiries can be directed to the corresponding author.

## Author contributions

LY, WY, TY, and ZC conceptualized and designed this study. QM, HZ, and SQ made contributions to the inclusion and exclusion criteria, search strategy and syntax, and data extraction and summary method. ZC and XL provided methodological advice and statistical expertise. WY and TY drafted the manuscript. LY supervised the work and polished the manuscript for important intellectual content to be published. All authors contributed to the article and approved the submitted version.
